# Humanization and Characterization of an Anti-Ricin Neutralization Monoclonal Antibody

**DOI:** 10.1371/journal.pone.0045595

**Published:** 2012-09-26

**Authors:** Wei-Gang Hu, Junfei Yin, Damon Chau, Laurel M. Negrych, John W. Cherwonogrodzky

**Affiliations:** Defence Research and Development Canada – Suffield, Medicine Hat, Alberta, Canada; The Scripps Research Institute, United States of America

## Abstract

Ricin is regarded as a high terrorist risk for the public due to its high toxicity and ease of production. Currently, there is no therapeutic or vaccine available against ricin. D9, a murine monoclonal antibody developed previously in our laboratory, can strongly neutralize ricin and is therefore a good candidate for humanization. Humanization of D9 variable regions was achieved by a complementarity-determining region grafting approach. The humanized D9 (hD9) variable regions were further grafted onto human heavy and light chain constant regions to assemble the complete antibody gene. A foot-and-mouth-disease virus-derived 2A self-processing sequence was introduced between heavy and light chain DNA sequences to cleave the recombinant protein into a functional full-length antibody molecule from a single open reading frame driven by a single promoter in an adenoviral vector. After expression in mammalian cells and purification, the hD9 was demonstrated to have equimolar expression of the full-length antibody heavy and light chains. More importantly, the hD9 exhibited high affinity to ricin with *K_D_* of 1.63 nM, comparable to its parental murine D9 (2.55 nM). In a mouse model, intraperitoneal (i.p.) administration of hD9, at a low dose of 5 µg per mouse, 4 hours after the i.p. challenge with 5×LD50 ricin was found to rescue 100% of the mice. In addition, administered 6 hours post-challenge, hD9 could still rescue 50% of the mice. The hD9 has the potential to be used for prophylactic or therapeutic purposes against ricin poisoning.

## Introduction

Ricin is a 60–65 kDa glycoprotein derived from castor beans [1]. It consists of a ricin toxin enzymatic-A (RTA) and a ricin toxin lectin-B (RTB) linked by a disulfide bond. RTB binding to galactose residues on cells triggers cellular uptake of the ricin [2] and facilitates transport of the RTA from endoplasmic reticulum to the cytosol [3], [4]. RTA enzymatically inactivates the ribosome to irreversibly inhibit protein synthesis [5]. A single molecule of RTA within the cell can completely inhibit protein synthesis, resulting in cell death. Ricin is one of the most potent toxins known for humans, with an LD50 of 1–20 mg/kg body weight when ingested and 1–20 µg/kg when inhaled or injected [6]. Due to its ease of production, worldwide availability, relative stability, and extreme lethality, ricin is listed as a Category B threat agent by Centers for Disease Control and Prevention (Atlanta, USA). There is currently no approved antidote available against ricin poisoning.

There are potentially two major groups of antidotes against toxins, antibodies and chemical compounds. The history of using antibodies as effective antidotes against toxins can be traced back to 1890 [7], when antiserum from a tetanus-immune animal protected tetanus toxin-mediated mortality of naïve animals. Since then, antibodies have played a pivotal role in neutralizing toxins [8], [9]. There are several advantages for antibodies as antidotes as compared to the chemical antidotes [10], [11], [12]. In the first place, antibodies have a long half-life in the body. Secondly, antibodies are natural products. Lastly, current biotechnology allows the development of antibodies possessing a defined specificity against most toxins.

Much work has been done on developing antibodies, both polyclonal and monoclonal, as antidotes against the toxin [13], [14], [15], [16], [17], [18], [19], [20]. Among these antibodies, one was a single chain variable fragment (ScFv) antibody developed from a non-human primate (*Macaca fascicularis*) and this ScFv antibody demonstrated a high sequence similarity (90%) to human counterparts. However, the rest of these antibodies were all derived from mice. While structurally similar, antibody sequence difference between mice and humans is sufficient to invoke an immune response in humans when murine antibodies were injected into humans. The immune response would result in a rapid removal of murine antibodies from the human blood, systemic inflammatory effects, and possible anaphylaxis, which can sometimes be fatal [21]. To overcome this hurdle, two murine anti-ricin mAbs were chimerized by genetically fusing murine antibody variable regions to human antibody constant regions to generate molecules with ∼70% human content [22], [23]. Chimeric antibodies successfully retained the mouse parental antibody antigen-binding specificity and diminished its immunogenicity. However, chimeric antibodies could still elicit an undesirable anti-variable region response [24]. As molecular biology technology developed, it became possible to further reduce the immunogenicity of the chimeric antibodies by replacing murine variable region frameworks (FRs) with those of the selected human antibodies using an approach called “complementarity-determined region (CDR) grafting” [25]. The resulting “humanized” antibodies contain 85–95% human sequences. Numerous clinical studies have confirmed that humanized antibodies are less immunogenic and more therapeutic than murine or chimeric antibodies in humans [26], [27]. However, the process of humanization of murine antibodies is much more challenging than constructing mouse-human chimeric antibodies. Humanization can result in a loss of activity.

In our previous study, a murine anti-ricin monoclonal antibody (mAb), D9 was found to be exceptionally effective in both pre- and post-exposure efficacy assays *in vivo*. Intraperitoneal (i.p.) administration of D9, at a low dose of 5 µg per mouse, 6 hours after or 6 weeks before the i.p. challenge with 5×LD50 ricin rescued 100% of the mice (manuscript submitted). These results indicate that mAb D9 is an excellent candidate to be developed as a potent antidote against ricin.

In this study, in order to humanize D9, the CDRs from the heavy and light chain (HC and LC) variable regions (VH and VL) forming antigen binding site of the murine mAb D9 were grafted onto antibody FRs from the selected donor human germline V and J genes of HC and LC. The humanized D9 (hD9) VH and VL were further grafted onto human HC and LC constant regions (CH and CL) to assemble the whole antibody gene. After expression in mammalian cells and purification, the recombinant hD9 (>95% human sequences) was demonstrated to retain antigen-binding specificity and therapeutic efficacy comparable to its parental murine D9. To the best of our knowledge, this is the first humanized anti-ricin mAb with demonstrated therapeutic efficacy *in vivo*. It has the potential for both prophylactic and therapeutic uses.

## Materials and Methods

### Ethics Statement

All mouse experiments were performed in strict accordance with the guidelines set out by the Canadian Council on Animal Care (CCAC). The animal care protocol was reviewed and approved by the Committee on the Ethics of Animal Experiments of Defence Research and Development Canada – Suffield (DRDC Suffield) (protocol number: J1C-10-1-1-0).

### Cloning of VH and VL Genes of D9

D9 VH and VL DNA sequences were determined as follows. Briefly, total RNA was isolated using a Qiagen RNeasy Plus Mini kit (Qiagen, Toronto, ON) from a D9 hybridoma cell line, made from female Balb/c mice in our laboratory (manuscript submitted). This was reverse-transcribed with Superscript II RNase H (Invitrogen, Burlington, ON) and an oligo dT primer (12–18 mer) to produce cDNA. Platinum Taq DNA Polymerase High Fidelity (Invitrogen) was used to amplify the VH and VL genes with the degenerate primers specific for mouse antibodies (Amersham Pharmacia Biotech, Baie d'Urfe, QB) by polymerase chain reaction (PCR) in an Eppendorf Mastercycler (Fisher Scientific, Nepean, ON). Distinct bands of ∼340 bp for VH and ∼325 bp for VL were detected on a 1.5% agarose gel after PCR. The bands were purified using a Qiagen Gel Extraction kit (Qiagen) and cloned into a Zero Blunt TOPO PCR cloning vector (Invitrogen) for sequencing.

### Molecular Modeling of D9 Variable Regions (Fv)

The homology modeling of D9 Fv was performed using PIGS (http://www.biocomputing.it/pigs), a web server for the automatic modeling of antibody Fv. When VH and VL protein sequences of D9 were entered, the sequences were analyzed by the PIGS server to search over a database of known antibody structures. The three-dimensional (3D) structure of D9 Fv was then constructed by homology modeling based on the structures of the VH (PDB No. 2NR6) of a mouse mAb against cockroach allergen Bla g 2 and the VL structure of a mAb against chicken egg-white lysozyme (PDB No. 1MLB). The final 3D structure of D9 was then visualized using a PDB molecular visualisation program, Deepview, for identification of the key FR residues, including Vernier Zone residues located within 5? of the CDRs, supporting CDR conformation and interchain packing residues located within 5? of VH-VL interface, contributing to VH-VL interaction. Meanwhile, the residue accessibility was calculated to determine their positions. The residue was defined as on the surface of antibody when it was greater than 30%.

### Humanization of Murine D9 Fv

The humanization of D9 Fv was done by CDR-grafting as described previously with minor modification [28]. Briefly, D9 CDR canonical structures were determined based on identification of unique residues both in CDRs and FRs using the online free program “AbCheck - Antibody Sequence Test” (http://www.bioinf.org.uk/abs/seqtest.html). In order to select human germline V genes as FR donors (FRs 1 to 3) for humanization of D9, the VH and VL amino acid sequences of D9 were separately subjected to IgBlast and IMGT searches against the entire human antibody germline V genes. Then human germline V genes with the same canonical structure as D9 were shortlisted. The human donor V genes were selected from the shortlist based on their highest CDR similarities (CDRs 1, 2 for HC V gene and CDRs 1–3 for LC V gene) to those of D9 VH and VL with consideration of key FR residue similarity. Human donor J genes to provide FR 4 for humanization of D9 were selected respectively based on their highest similarities to D9 VH and VL CDR3 and key FR residues in FR3. Finally, CDRs of D9 VH and VL were grafted onto the FRs of the selected human germline donor V and J genes respectively, resulting in humanized D9 (hD9) Fv. Furthermore, the hD9 VH and VL were grafted onto human gamma 1 HC CHs and kappa 1 LC CL, respectively, to assemble the complete humanized antibody gene, resulting in the full-length humanized antibody, hD9.

### Construction, Expression and Purification of hD9

The hD9 full-length DNA sequence (2 kb) including a LC leader sequence, the humanized LC (VL+CL), foot-and-mouth-disease virus (FMDV)-derived 2A self-cleavage linker encoding APVKQTLNFDLLKLAGDVESNPGP, a human antibody gamma 1 HC leader sequence, and humanized HC (VH+CH1 to 3) was synthesized by GenScript Corporation (Scotch Plaines, NJ) and cloned into pUC57, resulting in pUC57-hD9. A recombinant adenovirus vector, pAdhD9 expressing hD9 was constructed and the recombinant hD9 was expressed in HEK 293 mammalian cells using the AdEasy system (Qbiogene, Carlsbad, CA) [29], [30]. The expressed recombinant hD9 was purified using ImmunoPure Protein (L) agarose gel (Pierce, Brockville, ON).

### Sodium Dodcyl Sulfate-polyacrylamide Gel Electrophoresis (SDS-PAGE) Analysis of hD9

Antibodies were separated by 10% NuPAGE Bis-Tris SDS-PAGE gels using an XCell SureLock Novex Mini-Cell System (Invitrogen). The bands were visualized by SimplyBlue safestain staining (Invitrogen). The molecular weights of the samples were estimated by comparison to the relative mobility values of standards of known molecular weights.

### Affinity Analysis

The affinities for antibodies binding to ricin were determined using a Surface Plasmon Resonance (SPR) biosensor, SensiQ Pioneer (ICx Technologies, Oklahoma, OK). Briefly, ricin (10 µg/mL) diluted in 10 mM acetate buffer pH 4.5 was first immobilized onto the COOH1 chip following the standard 1-ethyl-3-(3-dimethylpropyl)-carbodiimide (EDC) plus N-hydroxysuccinimide (NHS) (Sigma-Aldrich) coupling chemistry, resulting in 250 response units (RU) of ricin being immobilized. The system was operated at 25°C. Kinetic measurements were carried out by 2 min injection at a flow rate of 25 µl/min of serial dilutions of each mAb from 0 to 500 nM in 4-(2-hydroxyethyl)-1-piperazineethanesulfonic acid (HEPES)-buffered saline containing 3 mM ethylenediaminetetraacetic acid (EDTA), 150 mM NaCl and 0.05% Tween-20 and dissociation for 6 min. The ricin immobilized chip surface was regenerated by injection of 10 mM phosphoric acid for 120 seconds after each cycle. The data of dissociation (*koff*) and association (*kon*) rate constants were obtained with the SensiQ Qdat software, corrected by subtraction of the zero antibody concentration flow cell as well as zero ricin flow cell; values for the apparent equilibrium dissociation constant (*K_D_*) were calculated from the ratio of *koff* and *kon*.

### Preparation of Ricin Stock

Ricin was prepared from castor bean seeds in DRDC Suffield. One LD50 of ricin for mice was determined by the i.p. injection of a series of ricin dilution into mice. The mice were observed for 7 days. The amount of ricin for 1×LD50 delivered by the i.p. route for one 20–25 gram female Balb/c mouse was 0.215 µg; 5×LD50 was 1.075 µg, or about 1 µg. For 5×LD50 of ricin delivered by the i.p. route, mice died within 2 days. Our laboratory was inspected and approved by the American Animal and Plant Health Inspection Service/Centers for Disease Control and Prevention for select agent use of ricin.

### *In vivo* Protection Assay

Female Balb/c mice (6 week old, 20–25 g) were obtained from the pathogen-free mouse-breeding colony at DRDC Suffield, with the original breeding pairs purchased from Charles River Canada (St Constant, QC). For post-exposure therapeutic efficacy study, groups of 8 mice were given 5×LD50 of ricin per mouse and then 5 µg of hD9 per mouse both by the i.p. route to mice at 2, 4, or 6 hours post-ricin poisoning. The mice were observed for morbidity and mortality over one week.

### Determination of Half-life in Serum

To evaluate the half-life of hD9 or D9 in serum, groups of 4 mice were injected by the i.p. route with 5 µg/mouse of antibody in 100 µl phosphate buffered saline (PBS), and were bled from a superficial tail vein at 1, 7, 14, and 23 days post injection. hD9 or D9 concentrations in sera over time were then measured by the anti-ricin immunoassay. Briefly, 96-well Nunc Maxisorp immunoassay plates (Canadian Life Technologies, Burlington, ON) were coated with 100 µl per well of 2.5 µg/ml ricin in carbonate bicarbonate buffer, pH 9.6, then incubated overnight at 4°C. After blocking with SuperBlock blocking buffer (Fisher Canada, Nepean, ON), the plates were incubated with 100 µl of serum dilutions for 2 hours at room temperature. Anti-ricin mAbs were detected by incubation with 1∶3,000 diluted horseradish peroxidase (HRP)-goat anti-mouse IgG (Jackson ImmunoResearch Laboratories, West Grove, PA) for D9 and 1∶5,000 diluted HRP-rabbit anti-human IgG (Jackson ImmunoResearch Laboratories) for hD9 followed by the addition of tetramethylbenzidine (TMB) (Kirkegaard and Perry Laboratories, Gathersburg, MD). Absorbance was measured at 615 nm by a microplate autoreader (Molecular Devices, Sunnyvale, CA). The data were expressed as percentages of the hD9 or D9 concentration in sera on day 1, and then plotted against time in days post treatment.

## Results

### Molecular Modeling and Structural Analysis of D9 Fv

**Figure 1:**
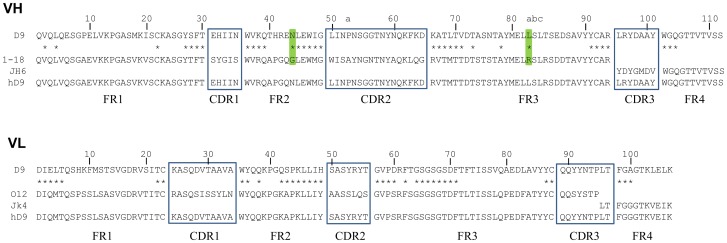
Humanization of D9 Fv by CDR-grafting. Residues are numbered according to Kabat. CDRs are marked with boxes and FRs are between boxes. Key FR residues are marked with *. Two key FR residues in D9 VH, which were kept in hD9 VH are marked with green.

**Figure 2:**
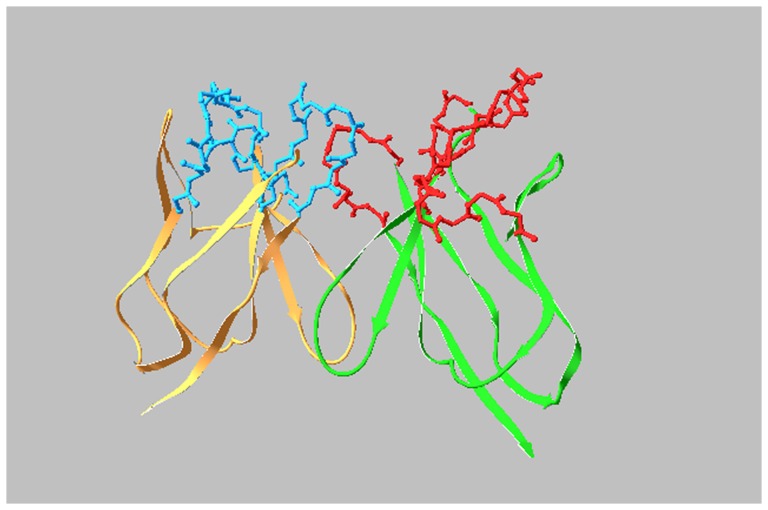
Molecular model of D9 Fv. FRs in VH and VL are shown in green and orange. CDRs in VH and VL are shown in red and blue.

D9 VH and VL mRNA was extracted from D9 hybridoma cells, amplified by Reverse Transcription-PCR and then sequenced. The deduced amino acid sequences are shown in Fig.1. The definition and numbering of CDRs were based on the Kabat scheme [31]. The CDR canonical structures for VH and VL were determined based on identification of unique residues both in CDRs and FRs as 2-1 and 1-1-2 [32]. The molecular model of D9 Fv was established through PIGS (http://www.biocomputing.it/pigs) based on the most homologous antibody VH (2NR6), sharing 86% identity and VL (1MLB), sharing 70% identity with the corresponding VH and VL of D9 in the database of known antibody structure. 3D structure of D9 was then visualized using a protein data bank (PDB) molecular visualisation program, Deepview, as shown in Fig.2. The potential key FR residues, such as Vernier Zone residues contacting CDRs and interchain packing residues affecting VH-VL interface, were identified (Fig.1).

### Humanization of D9

In order to select human germline antibody V genes as FR donors for D9 humanization, a shortlist of human germline antibody HC or LC V gene candidates with the same CDR canonical structure as the D9 counterpart was then formed to ensure that the human antibody donor FRs support the mouse CDR canonical structures (Table 1 or 2). Within the shortlist of human germline V genes, those with the highest similarity in CDRs (CDRs 1, 2 for HC and CDRs 1 to 3 for LC) and key residues of FRs 1 to 3 to the D9 counterparts were chosen as FRs 1–3 donors. As a result, human germline genes 1–18 and O12 were chosen for HC and LC respectively.

**Table 1:** Sequence alignment of CDRs 1–2 and FRs 1–3 from D9 VH with human germline HC V genes.

	FR1	CDR1	FR2	CDR2	FR3
	·········10········20········30	·····	····40········	50·a·······60····	····70········80·abc·······90···
	·········|·········|·········|	·····	····|·········	|··········|·····	····|·········|············|····
D9	QVQLQESGPELVKPGASMKISCKASGYSFT	EHIIN	WVKQTHRENLEWIG	LINPNSGGTNYNQKFKD	KATLTVDTASNTAYMELLSLTSEDSAVYYCAR
	·*?*·················*····****	·····	****····******	·················	******·*····*····*··········****
1–18	QVQLVQSGAEVKKPGASVKVSCKASGYTFT	SYGIS	WVRQAPGQGLEWMG	WISAYNGNTNYAQKLQG	RVTMTTDTSTSTAYMELRSLRSDDTAVYYCAR
1–69	QVQLVQSGAEVKKPGSSVKVSCKASGGTFS	SYAIS	WVRQAPGQGLEWMG	GIIPIFGTANYAQKFQG	RVTITADESTSTAYMELSSLRSEDTAVYYCAR
1-e	QVQLVQSGAEVKKPGSSVKVSCKASGGTFS	SYAIS	WVRQAPGQGLEWMG	GIIPIFGTANYAQKFQG	RVTITADKSTSTAYMELSSLRSEDTAVYYCAR
1-f	EVQLVQSGAEVKKPGATVKISCKVSGYTFT	DYYMH	WVQQAPGKGLEWMG	LVDPEDGETIYAEKFQG	RVTITADTSTDTAYMELSSLRSEDTAVYYCAT
5–51	EVQLVQSGAEVKKPGESLKISCKGSGYSFT	SYWIG	WVRQMPGKGLEWMG	IIYPGDSDTRYSPSFQG	QVTISADKSISTAYLQWSSLKASDTAMYYCAR
5-a	EVQLVQSGAEVKKPGESLRISCKGSGYSFT	SYWIS	WVRQMPGKGLEWMG	RIDPSDSYTNYSPSFQG	HVTISADKSISTAYLQWSSLKASDTAMYYCAR
7–4·1	QVQLVQSGSELKKPGASVKVSCKASGYTFT	SYAMN	WVRQAPGQGLEWMG	WINTNTGNPTYAQGFTG	RFVFSLDTSVSTAYLQICSLKAEDTAVYYCAR

Note: Key residues in FRs are marked with *.

**Table 2:** Sequence alignment of CDRs 1–2 and FRs 1–3 from D9 VL with human germline LC V genes.

	FR1	CDR1	FR2	CDR2	FR3	CDR3
	·········10········20··	······30···	·····40········	50·····	···60········70········80·······	·90····
	·········|·········|···	······|····	·····|·········	|······	···|·········|·········|········	·|·····
D9	DIELTQSHKFMSTSVGDRVSITC	KASQDVTAAVA	WYQQKPGQSPKLLIH	SASYRYT	GVPDRFTGSGSGSDFTFTISSVQAEDLAVYYC	QQYYNTP
	*****················**	···········	**·*···********	·······	****·*·********···············**	·······
O12	DIQMTQSPSSLSASVGDRVTITC	RASQSISSYLN	WYQQKPGKAPKLLIY	AASSLQS	GVPSRFSGSGSGTDFTLTISSLQPEDFATYYC	QQSYSTP
O2	DIQMTQSPSSLSASVGDRVTITC	RASQSISSYLN	WYQQKPGKAPKLLIY	AASSLQS	GVPSRFSGSGSGTDFTLTISSLQPEDFATYYC	QQSYSTP
O18	DIQMTQSPSSLSASVGDRVTITC	QASQDISNYLN	WYQQKPGKAPKLLIY	DASNLET	GVPSRFSGSGSGTDFTFTISSLQPEDIATYYC	QQYDNLP
O8	DIQMTQSPSSLSASVGDRVTITC	QASQDISNYLN	WYQQKPGKAPKLLIY	DASNLET	GVPSRFSGSGSGTDFTFTISSLQPEDIATYYC	QQYDNLP
A20	DIQMTQSPSSLSASVGDRVTITC	RASQGISNYLA	WYQQKPGKVPKLLIY	AASTLQS	GVPSRFSGSGSGTDFTLTISSLQPEDVATYYC	QKYNSAP
A30	DIQMTQSPSSLSASVGDRVTITC	RASQGIRNDLG	WYQQKPGKAPKRLIY	AASSLQS	GVPSRFSGSGSGTEFTLTISSLQPEDFATYYC	LQHNSYP
L14	NIQMTQSPSAMSASVGDRVTITC	RARQGISNYLA	WFQQKPGKVPKHLIY	AASSLQS	GVPSRFSGSGSGTEFTLTISSLQPEDFATYYC	LQHNSYP
L1	DIQMTQSPSSLSASVGDRVTITC	RASQGISNYLA	WFQQKPGKAPKSLIY	AASSLQS	GVPSRFSGSGSGTDFTLTISSLQPEDFATYYC	QQYNSYP
L15	DIQMTQSPSSLSASVGDRVTITC	RASQGISSWLA	WYQQKPEKAPKSLIY	AASSLQS	GVPSRFSGSGSGTDFTLTISSLQPEDFATYYC	QQYNSYP
L4	AIQLTQSPSSLSASVGDRVTITC	RASQGISSALA	WYQQKPGKAPKLLIY	DASSLES	GVPSRFSGSGSGTDFTLTISSLQPEDFATYYC	QQFNSYP
L18	AIQLTQSPSSLSASVGDRVTITC	RASQGISSALA	WYQQKPGKAPKLLIY	DASSLES	GVPSRFSGSGSGTDFTLTISSLQPEDFATYYC	QQFNSYP
L5	DIQMTQSPSSVSASVGDRVTITC	RASQGISSWLA	WYQQKPGKAPKLLIY	AASSLQS	GVPSRFSGSGSGTDFTLTISSLQPEDFATYYC	QQANSFP
L19	DIQMTQSPSSVSASVGDRVTITC	RASQGISSWLA	WYQQKPGKAPKLLIY	AASSLQS	GVPSRFSGSGSGTDFTLTISSLQPEDFATYYC	QQANSFP
L8	DIQLTQSPSFLSASVGDRVTITC	RASQGISSYLA	WYQQKPGKAPKLLIY	AASTLQS	GVPSRFSGSGSGTEFTLTISSLQPEDFATYYC	QQLNSYP
L23	AIRMTQSPFSLSASVGDRVTITC	WASQGISSYLA	WYQQKPAKAPKLFIY	YASSLQS	GVPSRFSGSGSGTDYTLTISSLQPEDFATYYC	QQYYSTP
L9	AIRMTQSPSSFSASTGDRVTITC	RASQGISSYLA	WYQQKPGKAPKLLIY	AASTLQS	GVPSRFSGSGSGTDFTLTISCLQSEDFATYYC	QQYYSYP
L11	AIQMTQSPSSLSASVGDRVTITC	RASQGIRNDLG	WYQQKPGKAPKLLIY	AASSLQS	GVPSRFSGSGSGTDFTLTISSLQPEDFATYYC	LQDYNYP
L12	DIQMTQSPSTLSASVGDRVTITC	RASQSISSWLA	WYQQKPGKAPKLLIY	DASSLES	GVPSRFSGSGSGTEFTLTISSLQPDDFATYYC	QQYNSYS
L2	EIVMTQSPATLSVSPGERATLSC	RASQSVSSNLA	WYQQKPGQAPRLLIY	GASTRAT	GIPARFSGSGSGTEFTLTISSLQSEDFAVYYC	QQYNNWP
L16	EIVMTQSPATLSVSPGERATLSC	RASQSVSSNLA	WYQQKPGQAPRLLIY	GASTRAT	GIPARFSGSGSGTEFTLTISSLQSEDFAVYYC	QQYNNWP
L6	EIVLTQSPATLSLSPGERATLSC	RASQSVSSYLA	WYQQKPGQAPRLLIY	DASNRAT	GIPARFSGSGSGTDFTLTISSLEPEDFAVYYC	QQRSNWP
L20	EIVLTQSPATLSLSPGERATLSC	RASQGVSSYLA	WYQQKPGQAPRLLIY	DASNRAT	GIPARFSGSGPGTDFTLTISSLEPEDFAVYYC	QQRSNWH
B2	ETTLTQSPAFMSATPGDKVNISC	KASQDIDDDMN	WYQQKPGEAAIFIIQ	EATTLVP	GIPPRFSGSGYGTDFTLTINNIESEDAAYYFC	LQHDNFP
A26	EIVLTQSPDFQSVTPKEKVTITC	RASQSIGSSLH	WYQQKPDQSPKLLIK	YASQSFS	GVPSRFSGSGSGTDFTLTINSLEAEDAATYYC	HQSSSLP
A10	EIVLTQSPDFQSVTPKEKVTITC	RASQSIGSSLH	WYQQKPDQSPKLLIK	YASQSFS	GVPSRFSGSGSGTDFTLTINSLEAEDAATYYC	HQSSSLP
A14	DVVMTQSPAFLSVTPGEKVTITC	QASEGIGNYLY	WYQQKPDQAPKLLIK	YASQSIS	GVPSRFSGSGSGTDFTFTISSLEAEDAATYYC	QQGNKHP

Note: Key residues in FRs are marked with *.

To select human germline antibody J genes as FR4 donors, CDR3 and FR4 sequences from D9 VH or VL were aligned with human germline JH or JK genes (Table 3 or 4). As shown in Table 3, JH genes encode a variable number of residues of VH CDR3. All human JH genes encode WGQ at the three key residue positions of FR4, but only JH4 and JH6 have a residue match with D9 CDR3. JH6 was chosen due to its higher similarity in FR4 to D9 than JH4. Differently, JK genes unanimously encode 2 residues of VL CDR3. As evident in Table 4, JK4 was chosen as the highest similarity to the D9 in terms of CDR3 and key residues in FR4.

**Table 3:** Sequence alignment of CDR3 and FR4 from D9 VH with human JH genes.

J gene	CDR3	FR4
		***········
D9	LRYDAAY	WGQGTTVTVSS
JH1	–-AEYFQH	WGQGTLVTVSS
JH2	–-YWYFDL	WGRGTLVTVSS
JH3	––-AFDI	WGQGTMVTVSS
JH4	––-YFDY	WGQGTLVTVSS
JH5	––NWFDP	WGQGTLVTVSS
JH6	YYYYYGMDV	WGQGTTVTVSS

Note: Key residues of FR4 are marked with *.

**Table 4:** Sequence alignment of CDR3 and FR4 from D9 VL with human JK genes.

J gene	CDR3	FR4
		***·······
D9	QQYYNTPLT	FGAGTKLELK
JK1	WT	FGQGTKVEIK
JK2	YT	FGQGTKLEIK
JK3	FT	FGPGTKVDIK
JK4	LT	FGGGTKVEIK
JK5	IT	FGQGTRLEIK

Note: Key residues of FR4 are marked with *.

**Figure 3:**
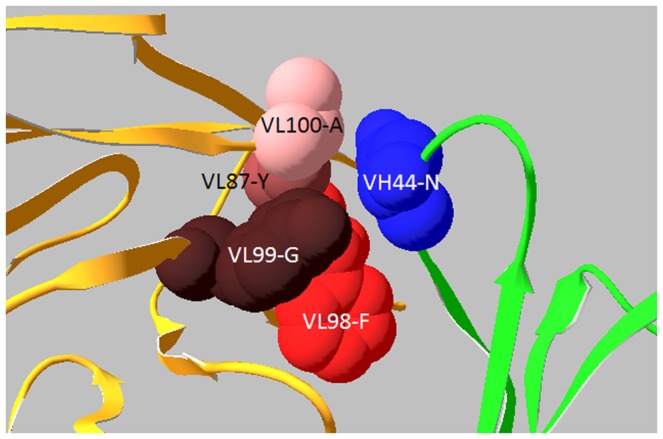
VH44-N interaction with four VL residues in VH-VL interface. VH44-N was an interchain packing residue, located in the VH-VL interface, interacting with VL87-Y, VL98-F, VL99-G, and VL 100-A. VH and VL are shown in green and orange.

**Figure 4:**
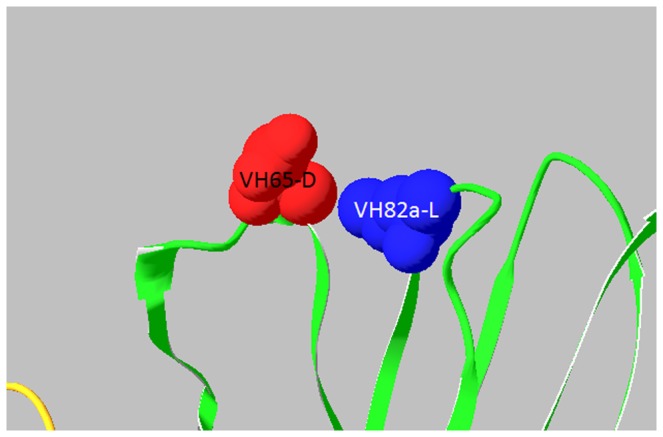
VL82a-L interaction with VH65-D of VH CDR2. VH82a-L was a Vernier Zone residue to support VH CDR2 by interacting with VH65-D of VH CDR2. VH and VL are shown in green and orange.

Consequently, human HC germline genes 1–18 and JH6 were selected as FR donors for humanization of D9 VH; human LC germline genes O12 and JK4 were selected as FR donors for humanization of D9 VL shown in Fig. 1. Seventy-five percent of the key FR residues of D9 was the same as human donors. Another 22% were different between murine D9 and human donors, but these were conservative (favor or neutral) substitutions in the same groups of amino acids shown in Table 5. The remaining 3% (2 residues) were not conserved (disfavored), these being VH44 (mouse N versus human G) and VH82a (mouse L versus human R). Most importantly, VH44-N was an unusual interchain packing residue, located in the VH-VL interface. Only 0.3% VH have N in the position 44, indicating it came from somatic mutation. As shown in Fig.3, within 5 ? of VH44-N there were four residues from VL (VL87-Y, VL98-F, VL99-G, and VL100-A), indicating that VH44-N interacted with these four VL residues in the VH-VL interface to make a contribution to the appropriate association between VH and VL. VH82a-L was a Vernier Zone residue to support CDR. Within 5 ? of VH82a-L there was only one residue from CDR, that was, VH65-D from VH CDR2, indicating that VH82a-L interacted with VH65-D to make a contribution to support VH CDR2 as shown in Fig. 4. Advantageously, molecular modeling revealed both of these as not solvent accessible, indicating these are not located on the surface of Fv and might not elicit an immune response in humans. Therefore, when the CDRs of D9 were grafted onto the donor human antibody FRs, VH44-N and VH82a-L were kept in hD9 Fv.

**Table 5:** Different key residues of FRs between human donors and D9.

Position		D9	Human donors	Favored or neutral substitution
VH	28	S	T	Yes (small, polar)
	38	K	R	Yes (polar, positive)
	44	N	G	No
	48	I	M	Yes (polar)
	66	K	R	Yes (polar, positive)
	67	A	V	Yes (polar)
	69	L	M	Yes (polar)
	71	V	T	Yes (polar)
	82a	L	R	No
VL	3	E	Q	Yes (polar)
	4	L	M	Yes (non-polar)
	42	Q	K	Yes (polar)
	43	S	A	Yes (tiny)
	49	H	Y	Yes (polar, aromatic)
	60	D	S	Yes (small, polar)
	69	S	T	Yes (small, polar)
	100	A	G	Yes (tiny, non-polar)

The VH of hD9 was further grafted onto the human gamma 1 HC CHs to form a complete HC, while the VL was grafted onto the human kappa 1 LC CL to form a whole LC.

### Construction and Expression of hD9

In order to obtain an equi-molar expression of antibody HC and LC in a single open reading frame (ORF) driven by one single promoter, a 2A self-cleavage linker encoding a 24-residue-oligopeptide, was introduced between HC and LC DNA sequences to express a full-length antibody from a ORF driven by a single promoter in an adenoviral vector [29], [30]. To get the expressed hD9 to be secreted to culture media, a leader sequence was added upstream of the HC and LC, respectively. The whole DNA sequence including the human antibody kappa LC O12 leader sequence, the humanized LC (VL+CL), 2A linker, 1–18 HC leader sequence, and humanized HC (VH+CH1+CH2+CH3), around 2 kb was synthesized and then cloned into an adenoviral vector for expression.

**Figure 5:**
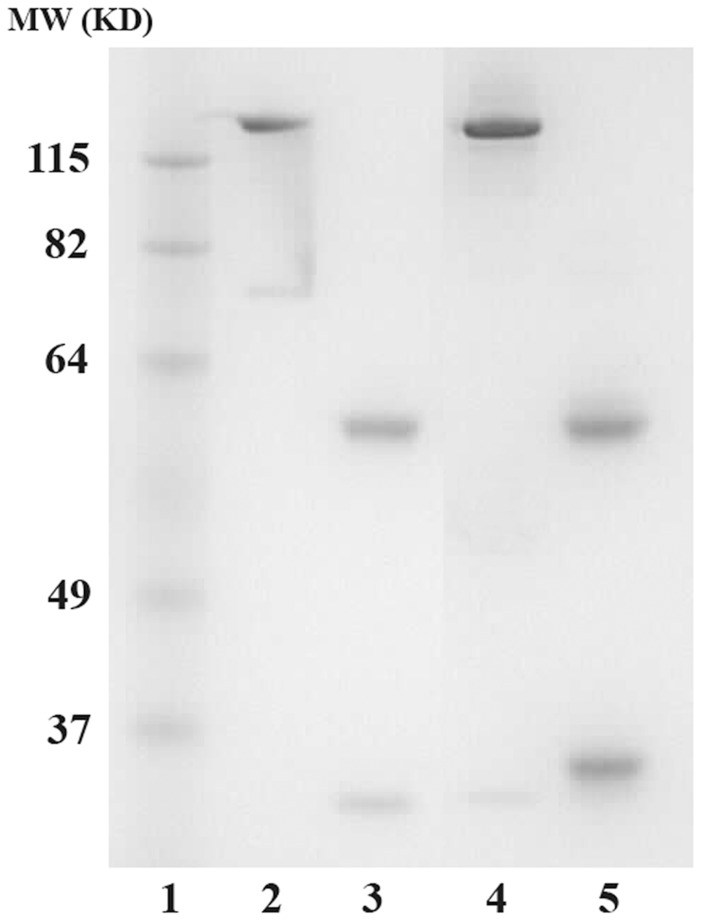
SDS-PAGE analysis of recombinant hD9. Lane 1 is a molecular marker. Lanes 2 and 4 are control human IgG1 and hD9 in non-reducing condition. Lanes 3 and 5 are control human IgG1 and hD9 in reducing condition.

The recombinant hD9 was expressed in HEK-293 cells and purified using an ImmunoPure Protein (L) agarose column. The purified product was subjected to 10% SDS-PAGE, and one prominent band of ∼150 kDa in non-reducing conditions, and two clear bands of ∼50 kDa and ∼25 kDa in reducing conditions (cleavage of disulfide bridges) were observed (Fig.5).

### Antigen-binding Affinity Analysis of hD9

**Figure 6:**
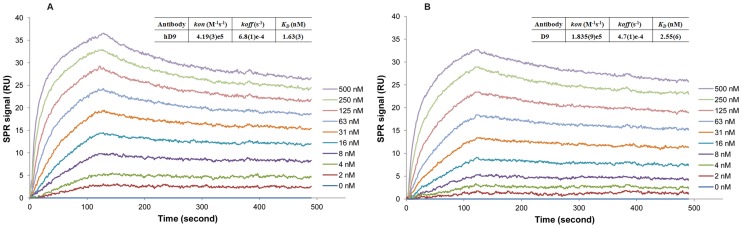
*In vitro* binding affinity analysis by SPR. SPR sensorgram of the kinetics of association and dissociation of a range of concentrations from 0 to 500 nm of hD9 (A) or D9 (B) to immobilized ricin.

In order to compare the antigen-binding affinity between hD9 and parental murine D9, measurements of affinity constant (*K_D_*) for antibodies binding to ricin were performed by SPR. Ricin was captured on a biosensor chip, various concentrations of hD9 or D9 were passed through the chip. The kinetics of association and dissociation were recorded in SPR sensorgram (Fig.6). The kinetic rate constants *kon* and *koff* were calculated from the ascending rate of resonance units during association and the descending rate during dissociation. The *K_D_* of hD9 or D9 for ricin was determined from the ratio of *koff/kon*. As shown in Fig.6, hD9 had high affinity to ricin with *K_D_* of 1.63 nM, comparable to D9 (2.55 nM).

### Therapeutic Efficacy Evaluation of hD9 *in vivo*

**Figure 7:**
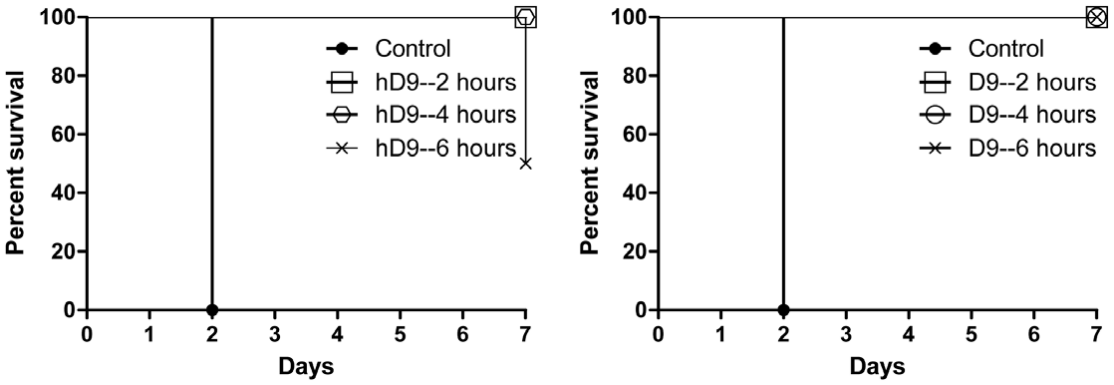
*In vivo* post-exposure therapeutic protection assay. Ricin was given at the dose of 5×LD50 to mice by i.p route. hD9 or D9 at the dose of 5 µg was administered i.p. at 2, 4, or 6 hours after ricin challenge and then mouse survival rate was monitored for 7 days.

To evaluate the therapeutic efficacy of hD9, a mouse model was used. Ricin was given at the dose of 5×LD50 to mice by i.p route and hD9 at the dose of 5 µg was administered i.p. at 2, 4, or 6 hours after ricin challenge. As shown in Fig.7, hD9 could rescue 100% of the mice up to 4 hours post-challenge, and at 6 hours post-challenge hD9 could still rescue 50% the mice, and extend the time to death of the other 50% of mice for up to 7 days in comparison with untreated controls which succumbed to ricin in 2 days. Its parental murine D9 showed 100% protection up to 6 hours post-challenge.

### Determination of hD9 Half-life in Mouse Serum

**Figure 8:**
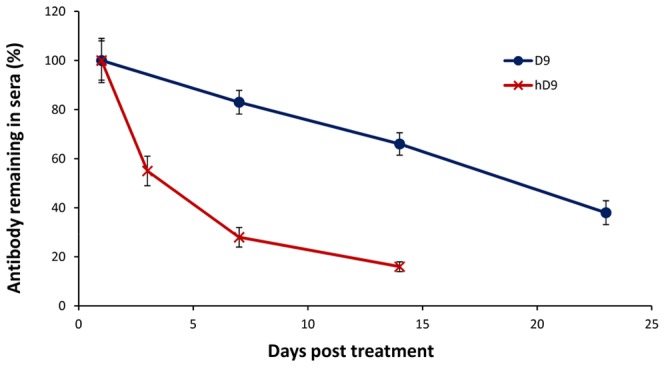
Half-life in mouse serum. hD9 or D9 at the dose of 5 µg was administered by the i.p. route into mice. Sera were collected at different time points to calculate hD9 or D9 concentrations using an immunoassay. The hD9 or D9 remaining in sera is expressed as percentages plotted against time in days on the figure.

To determine hD9 half-life in mouse serum, mice were injected i.p. with 5 µg of hD9 in 100 µl PBS. Mice were then bled from a superficial tail vein at specified time points. The quantity of antibody present in serum samples was determined by the immunoassay (Fig. 8). hD9 half-life was estimated as 3 days. On the other hand, when mice were treated with 5 µg D9/mouse, and D9 half life was around 19 days. It is understandable that a much higher decay rate of hD9 than D9 results from hD9’s significant portions of humanized and non-mouse molecule.

## Discussion

To date, most of anti-ricin neutralizing antibodies have been developed from mice. Only one was developed from macaques that share a high degree of antibody sequence similarity with humans [33]. This human-like antibody ScFv (43RCA) had 90% sequence similarity with human counterparts and demonstrated to neutralize ricin very effectively *in vitro* with a very high affinity (41 pM ) to RTA [15]. Murine antibodies have to be chimerized or humanized in order to reduce immune response against murine mAbs in humans. To date, only two murine antibodies have been chimerized [22], [23]. The humanization of murine antibodies, however, has not been reported yet. Different approaches have been developed to humanize murine mAbs [25], [28], [34], [35], [36]. Despite the development of molecular display technologies and transgenic animals for the generation of fully human antibodies, CDR grafting to transfer murine CDRs onto the human antibody FRs, remains an attractive and proven strategy for overcoming therapeutic deficiencies of murine antibodies.

There are two sources of human antibody sequences: mature (VH, VL) and germline (V, D, J). Mature sequences result from the recombination of germline genes V, D, and J for VH or V and J for VL [37]. The germline sequence has two advantages over the mature sequences as FR donors for murine CDR grafting. One is that these are less immunogenic, unlike the mature sequences that carry somatic mutations for affinity maturation generated by random processes, resulting in potential immunogenicity [38]. The other is increased flexibility [39], [40], resulting in more compatibility between murine CDRs and human FRs. Therefore, human germline sequences have increasingly been utilized as a source of FR donors. Since the D gene only encodes part of CDR3 for VH without any involvement of FR coding, it was not taken into account in the selection of germline genes as FR donors for humanization of D9 VH. In order to select human germline V and J gene candidates as FR donors for D9 humanization, D9 CDR canonical structures were determined first and then a shortlist, of germline human antibody V gene candidates sharing the same canonical structures with D9, was established to ensure that the human germline antibody V gene FRs support the mouse CDR canonical structures. Next, within the shortlist, those with highest similarity of CDRs and key FR residues to the D9 counterparts were chosen as the donors of FRs 1 to 3. For selection of human donors to provide FR4 for D9 humanization, human germline J genes were selected based on highest similarity to D9 CDR3 and key residues in FR4. Finally, the D9 CDRs were grafted onto these human donor FRs.

CDR-grafted antibodies tend to lose the parental binding affinity [41], [42]. The key for CDR grafted antibodies to retain the parental binding affinity lies in the preservation of the murine CDR conformation in the humanized antibody for antigen binding. The CDR conformation is mainly dependent on CDR canonical structures determined by a few canonical conserved residues located in CDRs and FRs [43], [44]. Furthermore, some key residues in FR fine-tune the CDR conformation. These include Vernier Zone residues, which form a layer underlying the CDRs [45] and interchain packing residues, which pair the CDRs of VH and VL [46]. Critical contribution of the Vernier Zone and appropriate association between VH and VL has been observed in antigen-binding affinity [45], [46], [47]. With the aid of computer modeling of D9, the Vernier Zone residues, located in 5? of the CDRs and the interchain packing residues, located in 5? of VH-VL interface were identified. Most of the key FR residues of D9 were the same or conserved as human donor antibodies. Only 2 residues were not conserved, VH44 (mouse N versus human G) and VH82a (mouse L versus human R). VH44-N was an unusual interchain packing residue, located in the VH-VL interface, interacting with VL87-Y, VL98-F, VL99-G, and VL100-A. Only 0.3% VH have N in the position 44, indicating it came from somatic mutation, which might indirectly enhance antibody binding. VH82a-L was a Vernier Zone residue to support VH CDR2 by interacting with VH65-D in VH CDR2. The substitution at both positions might significantly alter CDR’s conformation. As a result, these murine residues were retained. Advantageously, molecular modeling revealed both of these as not solvent accessible, indicating that these are not located on the surface of Fv and are unlikely to elicit an immune response in humans. Therefore, when the CDRs of D9 were grafted onto the donor human antibody FRs, VH44-N and VH82a-L were kept in hD9 Fv.

In order to have equimolar expression of HC and LC in mammalian cells, an FMDV-derived 2A self-cleavage linker was introduced between the two chain genes. The 2A oligopeptide sequence was expected to undergo self-cleavage to generate separate HC and LC after translation. The exact mechanism of 2A self-cleavage is still unknown. It has been hypothesized that the 2A sequence impairs peptide bond formation between 2A G and 2B P through a ribosomal skip mechanism [48]. Previously the 2A expression system has been successfully used to express multi-proteins in a single ORF including antibody HC and LC [49]. Meanwhile, leader sequences were added upstream to the HC and LC of hD9 gene to make hD9 secretable.

After the recombinant hD9 was expressed in mammalian cells, HC and LC appeared completely cleaved without detectable unpaired chains as shown in Fig.5. The cleavage was designed to occur at the C-terminus of the 2A sequence, leaving one residue to the N-terminus of the leader sequence of HC and 23 residues of 2A sequence fused to the end of LC, resulting in the LC with 27 kDa instead of 25 kDa (regular LC). Since the leader sequence is immediately cleaved from the HC once it has been translocated into the endoplasmic reticulum, the one extra residue would be removed with the leader sequence, leaving the HC without any extra residues. A potential drawback of this 2A expression system is that the small, 2A tag (23 residues) left at the end of the C-terminus of LC might affect antibody function or contribute to the antigenicity of antibodies. However, these problems have not been observed [50]. In this study, the recombinant hD9 was properly dimerized.

The specific binding activity of purified hD9 to ricin was evaluated. As shown in Fig.6, hD9 had high affinity to ricin with *K_D_* of 1.63 nM, comparable to D9 (2.55 nM), indicating humanization of D9 was successful.

hD9 appeared less efficacious than its parental murine D9. The difference is understandable, since hD9 had a much shorter half-life than D9 in mice. The half-life of hD9 was estimated to be around 3 days, compared to 19 days of D9 in mice. hD9 decayed in mice around 6 times faster than D9. There was only 25% of the hD9 left while still 85% of the D9 left in mice at day 7 post administration of antibodies, when 50% of the mice were dead in hD9-administrated group compared to no death in D9 group. Therefore, hD9 short half-life might be attributed to its slight lower efficacy than D9 in mice.

In conclusion, D9 was humanized by CDR-grafting to minimize murine antibody immunogenicity in humans. The resultant hD9 was expressed in mammalian cells and the recombinant hD9 was demonstrated to retain ricin-binding specificity and anti-ricin efficacy comparable to its parental murine D9. This hD9 has the potential utility for prophylactic or therapeutic purposes against ricin poisoning.
